# A Clinical Research on the Impact of Dexamethasone Versus Dexamethasone-Metoclopramide Combination in Reducing Postoperative Vomiting and Nausea After Cranial Surgery

**DOI:** 10.7759/cureus.15139

**Published:** 2021-05-20

**Authors:** Fahri Eryilmaz, Umar Farooque

**Affiliations:** 1 Neurological Surgery, Hittite University Corum Erol Olcok Training and Research Hospital, Corum, TUR; 2 Neurology, Dow University of Health Sciences, Karachi, PAK

**Keywords:** metoclopramide, dexamethasone, cranial surgery, nausea, neurosurgery, vomiting

## Abstract

Introduction

This brief study shows the consumption of two medications that are related to those patients who have gone through the complicated procedure of craniotomy. The basic aim of these drugs is to subside the after-effects of the procedure like postoperative nausea and vomiting in patients. Hereby, the study outlines the functional efficiency of dexamethasone along with the metoclopramide and dexamethasone alone.

Materials and methods

Randomly two groups were listed of 120 patients that have undergone elective craniotomy with ASA I-II. These groups were called out as group A and group B. Group A was under the medication of combination of dexamethasone and metoclopramide 8 mg and 10 mg, respectively, induced separately while group B was induced with 8 mg of dexamethasone along with 2 ml of normal saline. These drugs were induced right before anesthesia. The procedure from here on gets the same for both groups. After the surgical approach, a verbal evaluation was taken from the members of each group to collect specific data accordingly within the first 24 hours. As the method is double-blinded thus the patients were unaware of the ongoing research study. In any case of a mishap, rescue antiemetic drugs were also considered for the patients who would have experienced uncontrolled nausea and vomiting in the timeframe.

Results

The results show that only 16.7% of the patients from group A showed signs of nausea and only 5% showed vomiting while 31.7% of the patients from group B showed signs of nausea and 11.7% showed vomiting. It clearly showed that the patients tend to have either no sign of nausea and vomiting or showed little controllable nausea and vomiting when induced with dexamethasone and metoclopramide compared to those who were induced with the dexamethasone alone.

Conclusions

Postoperative nausea and vomiting are studied in terms of those who had undergone craniotomy. This study shows the prophylaxis of adverse effects of postoperative nausea and vomiting between the two groups under the influence of altered drugs. Thus, the results were noticeably in the favor of the combination treatment of dexamethasone and metoclopramide.

## Introduction

A craniotomy is a complicated procedure related to the neural function that can result in postoperative nausea and vomiting, moreover, this has been continued to be a cause of morbidity with adverse effects [1]. With the discoveries of the new antiemetic drugs, and modified anesthetic techniques followed by minimal invasiveness to surgical sites, the morbidity rate has been noticeably decreased [2]. Postoperative nausea and vomiting thus are the most commonly occurring after-effects of any surgical procedure and targets about 50% of the patients who have undergone craniotomy [3,4]. These adverse effects threaten life by dangerously elevating the arterial pressure and intracranial pressure of the patients [5]. All these contrary signs make the way for hemorrhage and ultimately for deaths. The minimal signs and symptoms associated with postoperative nausea and vomiting include dry mouth, sour taste, dehydration, electrolyte imbalance, restlessness, restricted mobilization, and fatigue giving more in-hospital phase to the patients and make a risk out of their health [6,7]. This study, discussed here, highlights all the necessary factors that tend to trigger postoperative nausea and vomiting after craniotomy and thus concludes the efficiency of the drug that helped to subside those adverse effects with less expenditure of time and health. So, in this scenario, the combination treatment tactic of dexamethasone and metoclopramide turned out to be more effective and useful.

## Materials and methods

This study was conducted at Dow University of Health Sciences, Karachi for a duration of one year from May 2019 to May 2020. It was approved by the Hospital Ethical Committee of Dow University of Health Sciences. It included 120 patients admitted to our hospital between the age of 33 and 65 years that were supposed to undergo craniotomy. This study is upon the method of a randomized clinical trial and was kept super confidential from the patients. All these patients were allocated into two groups A and B and were induced with drugs dexamethasone along with metoclopramide and dexamethasone alone, respectively. The standard dosage of 8 mg of dexamethasone and 10 mg of metoclopramide was given before anesthesia to group A, while group B only received 8 mg of dexamethasone. All the essentials were kept under strict monitoring before inducing anesthesia with 2 mg/kg of propofol and 0.1 mg/kg of nalbuphine. The surgical procedure then proceeded with a similar protocol for all. A muscle relaxant was given followed by three minutes of monitoring; later, an endotracheal tube was intubated. Ventilation was monitored and surgery was completed. At the end of the surgery, volatile agent and nitrous oxide were turned off; 30 mg of ketorolac was given for postoperative pain control, and 2.5 mg of neostigmine with 0.5 mg of glycopyrrolate was given for reversal of neuromuscular block, and the trachea was extubated on regaining spontaneous breathing and opening of the eyes. The smooth procedure of surgery was then followed by the strict evaluation for the first 24 hours and assessing any arising signs of postoperative nausea and vomiting by simple verbal cues from patients. Rescue antiemetic drug was given to the patients who experienced any nausea or unstoppable vomiting. It was easy to conduct the data of the patients as the majority from group A showed expected results of zero postoperative nausea and vomiting. However, the study is settled with the statistical data approach of the patients from both the group as qualitative variables of percentage. Other findings like age, weight, duration of surgery, surgical technique, and duration of anesthesia were analyzed by using student t-test, while gender, frequency, and intensity of nausea and vomiting and use of rescue antiemetic drugs were analyzed by using chi-square test. A p-value of less than 0.05 was considered significant. 

## Results

The results were taken free from any sort of bias concerning age, gender, weight, and physical status among the patients of the groups. The demographic features of the patients of both groups are shown in Table 1.

**Table 1:** The demographic features of the patients. kg: kilogram.

Variable	Group A	Group B	p-value
Age (years)	47±18	40±19	0.928
Sex (male:female)	33:27	31:29	0.851
Weight (kg)	57±19	52±17	0.605
Duration of surgery (minutes)	4±1.2	4±1.4	0.868
Duration of anesthesia (minutes)	17±5	17±6	0.462

A rough statistical data was designed to put forward the aim and use of the drugs. Among the members of group A, about 83.3% of the patients showed absolutely no sign of nausea, while 16.7% had nausea and only about 5% showed vomiting within the first observatory 24 hours. Similarly, when looking at the data of group B, about 68.3% of patients showed no sign of nausea and vomiting whereas about 31.7% of the patients had nausea, and an estimated 11.7% showed vomiting in the specified timeframe as shown in Table 2.

**Table 2:** Incidence of postoperative nausea and vomiting.

Time (hours)	Event	Group A	Group B	p-value
0–2	Nausea	7 (11.7%)	13 (21.7%)	
	Vomiting	3 (5%)	5 (8.3%)	
2–4	Nausea	5 (8.3%)	6 (10%)	
	Vomiting	0	4 (6.7%)	
4–24	Nausea	0	4 (6.7%)	
	Vomiting	0	0	
First 24	Nausea	10 (16.7%)	19 (31.7%)	0.042
	Vomiting	3 (5%)	7 (11.7%)	0.038

This undoubtedly concluded that the frequency of nausea and vomiting observed is lower in those who were under the combination treatment management than those who took dexamethasone alone. Emergency conditions where 4 mg of intravenous ondansetron was given to only three patients of group A while only about 11 patients needed it from group B as shown in Table 3. This elevates the use of rescue antiemetics for those who opt for the drugs given to the patients of group B.

**Table 3:** Use of rescue antiemetic drugs.

Group	No of patients	Percentage	p-value
A	3	5%	0.015
B	11	18%	

## Discussion

This clinical-based research carried on the age group of 33-65 years of patients undergoing craniotomy has shown a great impact on the use of appropriate drugs for subsiding the rising postoperative issues like nausea and vomiting. The comparable study highlights the efficiency of dexamethasone when given in combination with metoclopramide or alone. As dexamethasone has been effective against vomiting and nausea thus it is called an antiemetic drug [8]. However, earlier dexamethasone was taken by patients after chemotherapy to reduce the chances of motion sickness, vomiting, and nausea. A new study gained attention when steroids were used for these adverse effects [9]. Steroids tend to become a threat to immunity and due to their high-intensity dose, it was reconsidered when using as an antiemetic drug [10,11]. The incidence and frequency of postoperative nausea and vomiting desirably reduced when dexamethasone replaced it. It became a helpful asset to the medical world and gained attention. Glucocorticoids were also used for a similar purpose but gets in vain when other harmful effects started to raise [12,13]. Moreover, metoclopramide being a D2 receptor acting as a central dopaminergic antagonist increases gastric emptying. Looking into the mechanisms of action of the different drugs used for the same purpose, it is seen that for the treatment of postoperative nausea and vomiting, dexamethasone was found to be superior in the control of nausea and vomiting [5]. However, the results of the statistical calculation obtained from the study weightage the theories about the efficiency of dexamethasone against vomiting and nausea [14-16].

## Conclusions

The present clinical-based study demonstrated the statistical results of the combination treatment of drugs that include dexamethasone in addition to metoclopramide is superior to when dexamethasone is given alone in preventing postoperative nausea and vomiting in patients undergoing elective craniotomy, simplifying the complexity of neurosurgical health care facilities and adding more to life. Further studies on a larger scale are needed in the future to validate the results of this study.
